# Supplementary material for: Changes in semantic memory structure support successful problem-solving and analogical transfer
Source: Commun Psychol. 2024 Jun 7;2:54. doi: 10.1038/s44271-024-00100-w (PMC11332086; doi:10.1038/s44271-024-00100-w)
Supplement: Supplementary file 2 — Supplementary Information [file 44271_2024_100_MOESM2_ESM.pdf]

Changes in semantic memory structure support successful problem-solving and analogical transfer
-
Supplementary information

Théophile Bieth[1,2], Yoed N. Kenett[3], Marcela Ovando-Tellez[1], Alizée Lopez-Persem[1], Célia Lacaux[1,4], Marie Scuccimarra[1], Inès Maye[1], Jade Sénéchal[1], Delphine Oudiette[1,4]* & Emmanuelle Volle[1]*

Affiliation:
[1] Sorbonne University, Institut du Cerveau - Paris Brain Institute -ICM-, Inserm, CNRS, AP-HP Hôpital de la Pitié-Salpêtrière, Paris, France
[2] Neurology department, Pitié-Salpêtrière hospital, AP-HP, F-75013, Paris, France
[3] Faculty of Data and Decision Sciences, Technion – Israel Institute of Technology, Haifa 3200003 Israel
[4] Sleep center, Pitié-Salpêtrière hospital, AP-HP, F-75013, Paris, France

* These authors contributed equally

Corresponding authors:
Emmanuelle Volle (emmavolle@gmail.com)
Delphine Oudiette (delphine.oudiette@gmail.com)
Théophile Bieth (theo_bieth@hotmail.fr)

Supplementary Figures

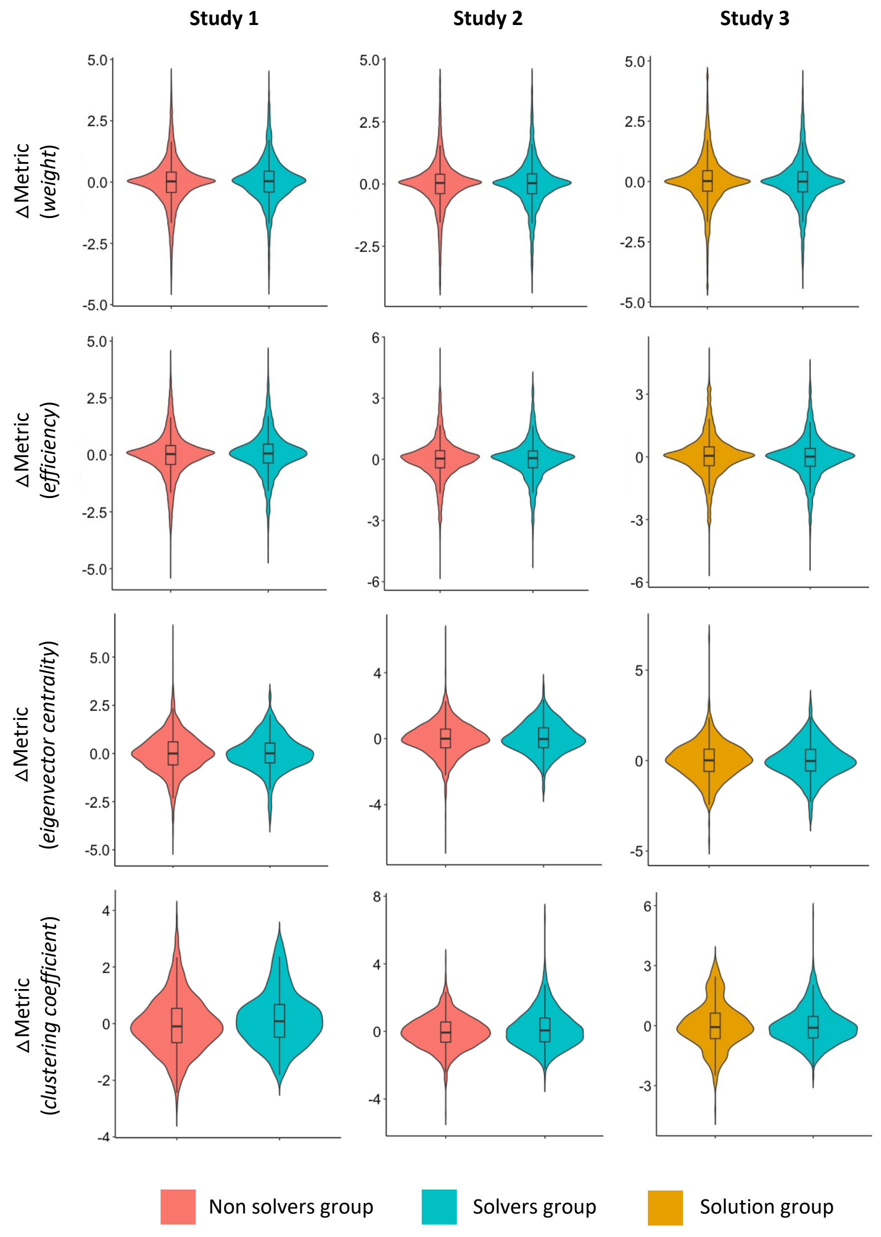

Figure S1. Distribution of ΔMetric. A. Distribution of ΔMetric for each metric (*weight*, *efficiency*, *eigenvector centrality*, *clustering coefficient*) for non solver (in red) and solver (in blue) groups (for Studies 1 and 2), and solver (in blue) and solution (in yellow) groups (for Study 3). Box plots indicate the upper and lower quartiles, and the black horizontal line within the boxes symbolizes the median.

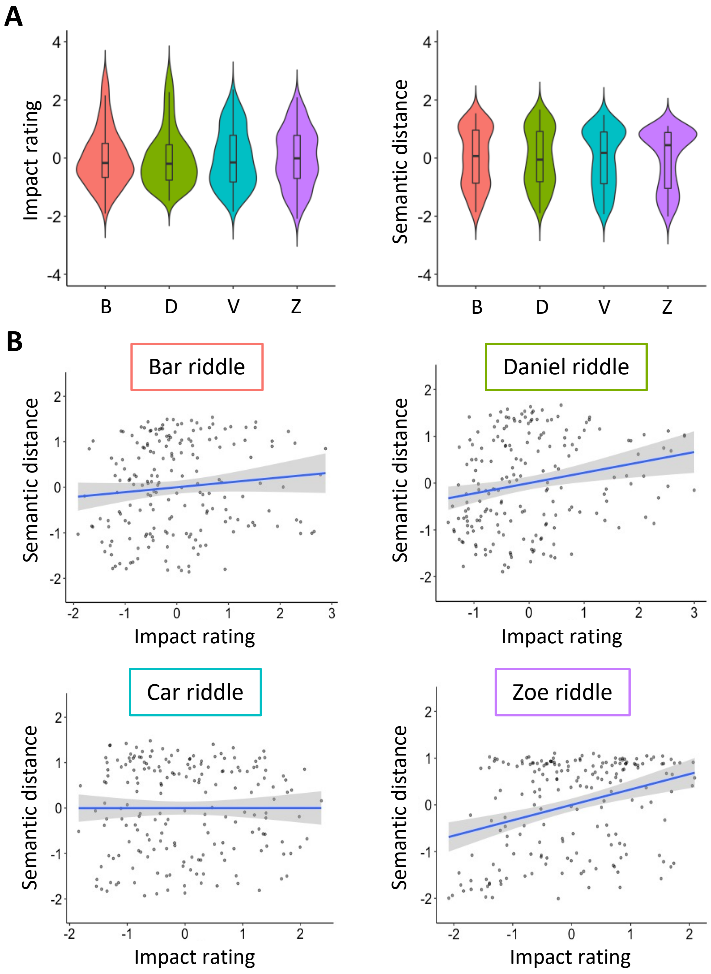

Figure S2. *Impact ratings* and *semantic distance* of riddles-related word pairs. A. Distribution of the *impact rating* (left) and *semantic distance* (right) for each word pair (i.e., edges n=190) for Bar (B, in red), Daniel (D, in green), Car (C, in blue), and Zoe (Z, in violin) riddles. Scores were normalized (z-scored) within each riddle. Box plots indicate the upper and lower quartiles, and the black horizontal line within the boxes symbolizes the median. B. *Semantic distance* as a function of *impact rating* for each riddle separately. Solid blue lines represent the linear regression between the two variables.

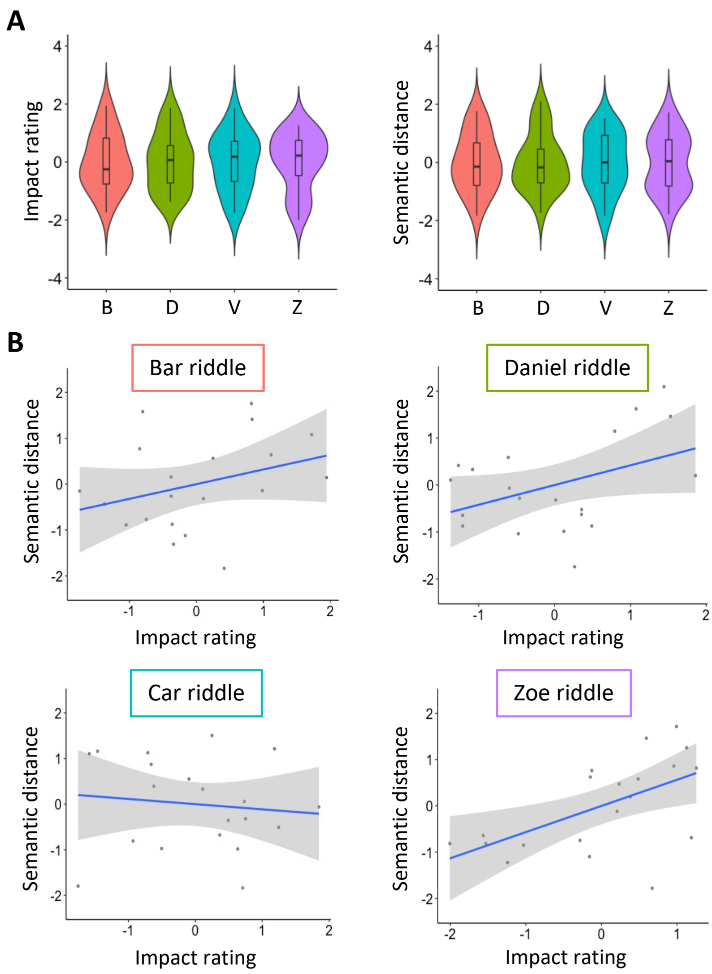

Figure S3. *Impact ratings* and *semantic distance* of riddles-related word. A. Distribution of the *impact rating* (left) and *semantic distance* (right) for each word (i.e., node, n=20) for Bar (B, in red), Daniel (D, in green), Car (C, in blue), and Zoe (Z, in violin) riddles. Scores were normalized (z-scored) within each riddle. Box plots indicate the upper and lower quartiles, and the black horizontal line within the boxes symbolizes the median. B. *Semantic distance* as a function of *impact rating* for each riddle separately. Solid blue lines represent the linear regression between the two variables.

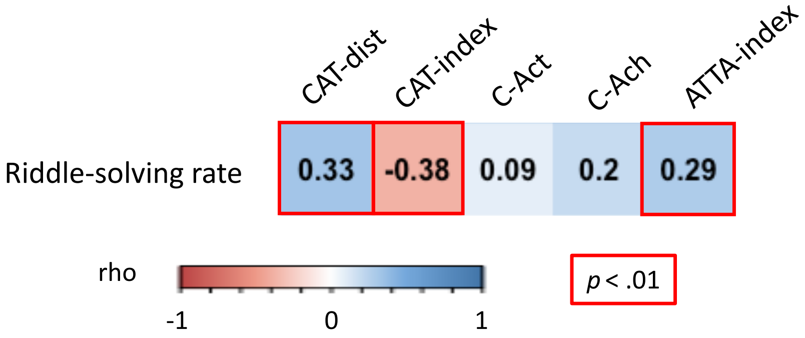

Figure S4. Correlation matrix between riddle-solving rate and creativity tasks. Correlation coefficients are indicated in the matrix (rho value, Spearman correlation) and with the color bar. Significant correlations after Bonferroni correction ($p < .01$) are highlighted with a red square. *CAT-dist* refers to the number of correct distant trials in the CAT and *CAT-index* to the difference in performance between close and distant trials (the lower the *CAT-index,* the higher creativity abilities). *C-Act* and *C-Ach* refer to the involvement in creative activities and achievements in the ICAA, respectively. *ATTA-index* refers to the final combined and scaled score of ATTA.

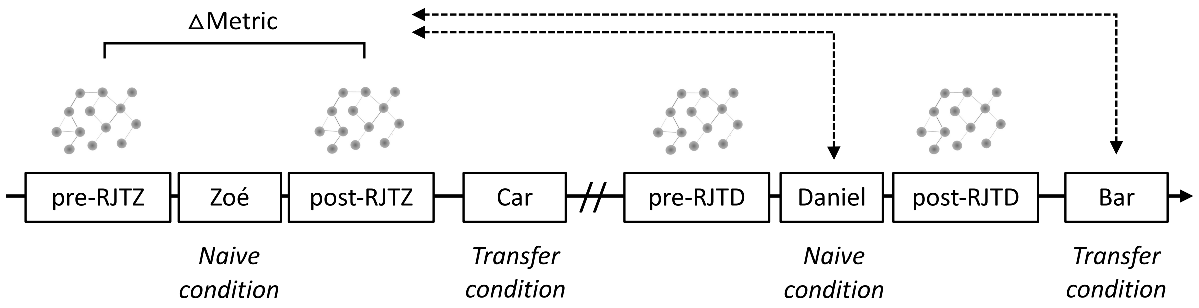

Figure S5. Schematic representation of our analyses. We investigate if restructuring related to a riddle (e.g., Zoe riddle) was associated with solving another riddle that did not have any analogical similarities (e.g., Daniel or Bar riddle).

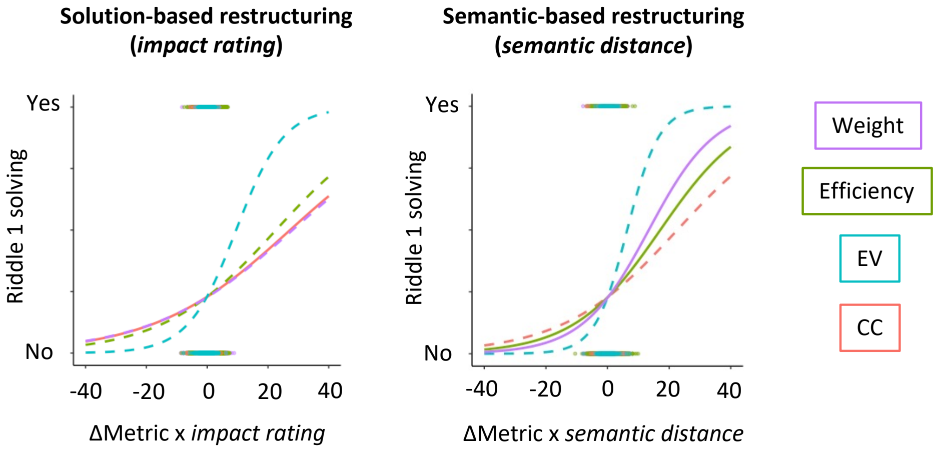

Figure S6. Local SemNet changes were associated with problem-solving (Study 2). Graphs represent the ΔMetric weighted by the *impact rating* (ΔMetric x *impact rating* – solution-based restructuring) or the ΔMetric weighted by the *semantic distance* (ΔMetric x *semantic distance* – remoteness-based restructuring) as a function of solving success. Lines represent the fitting curves of the interaction effect (ΔMetric x *impact rating* on the left, and ΔMetric x *semantic distance* on the right) in predicting problem-solving in the four mixed models computed for local SemNet metrics (*weight* – in purple, *efficiency* – in green, *eigenvector centrality* – EV in blue, and *clustering coefficient* – CC in red). A solid line indicates that a significant effect ($p < .05$) was found.

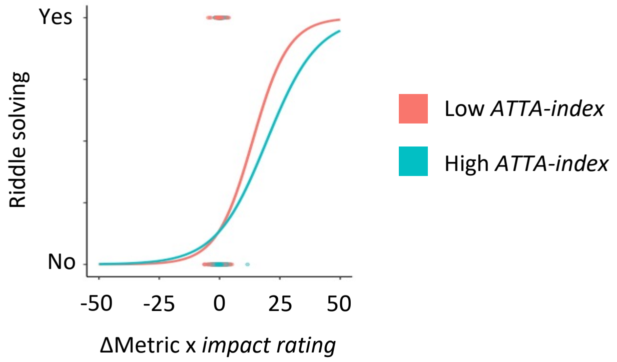

Figure S7. *Relationship between ATTA creativity measure, restructuring, and problem-solving*. Three-way interaction effect of ΔMetric, *impact rating*, and *semantic distance* on problem-solving for *eigenvector centrality*. Graphs represent the ΔMetric weighted by the *impact rating* (ΔMetric x *impact rating*) as a function of solving success for individuals with low *ATTA-index* (i.e., low divergent thinking abilities, in red) and high *ATTA-index* (i.e., high divergent thinking abilities, in blue). Lines represent the fitting curves of the interaction effect (ΔMetric x *impact)* in predicting problem-solving with the same color code.

Supplementary Tables

	Problem statement
Zoe riddle	
French	Zoé jette une pierre qui atterrit dans le ciel. Comment est-ce possible et dans quel contexte ?
English	Zoe throws a stone that lands in the sky. How is it possible and in which context?
Car riddle	
French	Un homme pousse sa voiture. Il s'arrête devant un hôtel et se sait alors totalement ruiné. Pourquoi ?
English	A man pushes his car in front of a hotel. Instantly he knows he's bankrupt. What's going on?
Bar riddle	
French	Un homme entre dans un bar et se dirige vers le comptoir pour demander un verre d'eau. Le barman sort un pistolet et le pointe vers l'homme. Ce dernier dit « Merci ! » et s'en va. Comment expliquer la situation ?
English	A man walks into a bar and ask for a glass of water. The bartender points a shotgun at the man. The man says "Thank you" and walks out. How to make sense of this story?
Daniel riddle	
French	Daniel croise dans la rue un couple qui marche vers lui. Alors qu'ils ne se connaissent pas, Daniel tape violement l'homme. Sa femme à ses côtés est paniquée. L'homme est sous le choc, mais Daniel est fier de lui. Pourquoi ?
English	Daniel meets a couple walking towards him in the street. Although they don't know each other, Daniel violently hits the man. His wife at his side is panicked. The man is in shock, but Daniel is proud of himself. Why?

Table S1. Riddle statements. Riddles used in our experiment with their related English translation. Solutions are provided in a separate table (Table S2).

	Problem response
Zoe riddle	
French	Zoé joue à la marelle
English	Zoe plays hopscotch
Car riddle	
French	L'homme joue au Monopoly
English	The man plays Monopoly
Bar riddle	
French	L'homme a le hoquet
English	The man has hiccups
Daniel riddle	
French	L'homme était en train de s'étouffer
English	The man was suffocating

Table S2. Riddle answers. Hopscotch is a famous backyard children's game in which players have to throw a stone in a square drawn on the ground and then hop on them. The last square is traditionally represented in France as the sky.

Zoe riddle		Car riddle		Bar riddle		Daniel riddle	
French	English	French	English	French	English	French	English
air	air	acheter	to buy	bar	bar	agression	attack
arrivée	arrival	arrêter	to stop/arrest	boire	to drink	amour	love
atterrir	to land	banque	bank	bruit	noise	bleu	blue
avion	plane	billet	bill	chargé	loaded	choc	shock
caillou	stone	carte	card	comptoir	counter	cœur	heart
chaussure	shoe	casino	casino	danger	danger	coup	hit
chiffre	number	chance	luck	détente	rest/trigger	couple	couple
ciel	sky	départ	departure	dispute	quarrel	crier	to yell
cour	yard	dormir	sleep	ivre	drunk	dispute	quarrel
dessin	drawing	emprunt	loan	menace	threat	dos	back
écrire	to write	fortune	fortune	merci	thanks	douleur	pain
espace	space	gare	station	mourir	to die	fou	mad
gravité	gravity	hôtel	hotel	peur	fear	gorge	throat
jardin	garden	paix	peace	pistolet	shotgun	jaloux	jealous
lancer	to throw	police	police	remède	remedy	rouge	red
pied	foot	prison	jail	soulagé	relieved	sang	blood
sauter	to jump	roue	wheel	surprise	surprise	secours	rescue
sol	ground	ruiné	ruined	sympa	friendly	soulager	to relieve
tracer	to trace	vacances	vacation	télé	television	tromper	to cheat
voler	to fly	voiture	car	verre d'eau	glass of water	violent	violent

Table S3. Riddle-specific word lists used in the RJT. Lists of 20 words preselected for each riddle with their respective English translation.

Riddle	Number of exclusions because participants already knew the riddle	Proportion of participants who solved the riddle	Number of exclusions because of SemNet cleaning or technical issues	Proportion of participants who solved the riddle after exclusions due to SemNet cleaning	
Zoe	0	14/49	13	10/36	n = 49
Daniel	0	4/49	15	3/34	
Bar	5	3/45	11	2/34	n = 50
Car	1	9/49	11	7/38	

Table S4. Sample size at the riddle level (Study 1).

	Number of participants for the behavioral analysis	Number of participants for the SemNet analyses
2 riddles solved	0	0
No riddle solved	66	45
1 solved and 1 not solved	28	17
1 solved (the other not analyzed*)	2	5
1 not solved (the other not analyzed*)	2	13
Total number of participants that were analyzed	98**	80

Table S5. Sample size at the individual level (Study 1).
* The other riddle was not analyzed because participants already knew the riddle or because its RJT was excluded after the SemNet cleaning.
** The total number of subjects was not 99 because one participant already knew the two riddles.

	Weight	Efficiency	Clustering coefficient	Eigenvector centrality
Weight	1.00	.87	.88	.52
Efficiency	.87	1.00	.64	.42
Clustering coefficient	.88	.64	1.00	.52
Eigenvector centrality	.52	.42	.52	1.00

Table S6. Correlation matrix between metrics. Pearson correlation was used. This matrix was used to compute the vector of eigenvalues required to calculate the effective number of tests.

Condition	Zoe riddle	Car riddle	Daniel riddle	Bar riddle	All riddles
Naive	14 (5) / 49<br>28.6%	9 (3) / 49<br>18.4%	4 (1) / 49<br>8.2%	3 (2) / 45<br>6.7%	30 (11) / 19<br>15.6%
Transfer	23 (17) / 50<br>46%	20 (17) / 47<br>43%	14 (8) / 50<br>28%	8 (7) / 44<br>18%	65 (49) / 191<br>34%

Table S7. Problem-solving. Solving rates are provided for each condition: *naive* condition (i.e., not preceded by an analogous riddle), and *transfer* condition (i.e., preceded by an analogous riddle). The total number of solvers (with the number of Eureka reports when solving the riddle in parentheses), over the total number of participants analyzed are indicated.

	Creative measure		β	SE	z-value	p-value	CI95%
Weight	CAT-dist	△Metric	.10	.11	.90	.37	-.11 .31
		IR	-.001	.03	-.07	.94	-.05 .05
		CAT-dist	-.03	.89	-.03	.97	-1.78 1.72
		△Metric x IR	.07	.03	2.56	**.01**	.02 .13
		△Metric x CAT-dist	-.12	.12	-1.06	.29	-.35 .11
		IR x CAT-dist	.02	.03	.55	.58	-.04 .07
		△Metric x IR x CAT-dist	-.02	.03	-.58	.56	-.07 .04
	CAT-index	△Metric	.11	.11	1.04	.30	-.10 .32
		IR	-.002	.03	-.06	.95	-.05 .05
		CAT-index	-.10	.90	-.12	.91	-1.88 1.67
		△Metric x IR	.07	.03	2.51	**.012**	.02 .13
		△Metric x CAT-index	.14	.10	1.35	.18	-.06 .34
		IR x CAT-index	-.01	.03	-.44	.66	-.06 .04
		△Metric x IR x CAT-index	.03	.03	1.17	.24	-.02 .09
	ATTA-index	△Metric	.09	.11	.80	.43	-.13 .30
		IR	-.008	.003	-.03	.98	-.05 .05
		ATTA-index	.08	.93	.08	.93	-1.74 1.90
		△Metric x IR	.07	.03	2.57	**.01**	.02 .13
		△Metric x ATTA-index	-.10	.11	-.94	.35	-.31 .11
		IR x ATTA-index	.01	.03	.53	.59	-.04 .06
		△Metric x IR x ATTA-index	.02	.03	.66	.51	-.04 .07
Efficiency	CAT-dist	△Metric	.12	.11	1.58	.25	-.09 .33
		IR	.004	.03	.16	.87	-.05 .05
		CAT-dist	-.02	.89	-.02	.98	-1.77 1.72
		△Metric x IR	.08	.03	2.92	**.004**	.03 .14
		△Metric x CAT-dist	-.16	.12	-1.36	.17	-.39 .07
		IR x CAT-dist	.002	.03	.07	.95	-.05 .06
		△Metric x IR x CAT-dist	-.02	.03	-.66	.51	-.08 .04
	CAT-index	△Metric	.13	.11	1.23	.22	-.08 .35
		IR	.01	.03	.22	.82	-.05 .06
		CAT-index	-.10	.91	-.11	.91	-1.88 1.67
		△Metric x IR	.08	.03	2.99	**.003**	.03 .14
		△Metric x CAT-index	.13	.10	1.21	.23	-.08 .33

		IR x CAT-index	.003	.03	.14	.89	-.05	.05
		△Metric x IR x CAT-index	.05	.03	1.86	.06	-.003	.11
	ATTA-index	△Metric	.11	.11	1.06	.29	-.10	.32
		IR	.005	.03	.18	.85	-.05	.05
		ATTA-index	.08	.92	.08	.93	-1.73	1.89
		△Metric x IR	.08	.03	2.91	**.004**	.03	.14
		△Metric x ATTA-index	-.13	.10	-1.26	.21	-.34	.07
		IR x ATTA-index	-.005	.03	-.20	.84	-.05	.04
		△Metric x IR x ATTA-index	.01	.03	.43	.67	-.04	.06
Eigenvector centrality	CAT-dist	△Metric	.002	.09	.03	.98	-.17	.17
		IR	.01	.08	.15	.88	-.14	.17
		CAT-dist	-.05	.79	-.07	.95	-1.59	1.49
		△Metric x IR	.28	.09	3.11	**.002**	.11	.46
		△Metric x CAT-dist	.02	.10	.24	.81	-.17	.22
		IR x CAT-dist	-.006	.09	-.06	.95	-.18	.17
		△Metric x IR x CAT-dist	.05	.10	.52	.60	-.15	.25
		△Metric						
	CAT-index	△Metric	.003	.09	.03	.98	-.17	.17
		IR	.01	.08	.12	.90	-.15	.17
		CAT-index	-.12	.79	-.15	.88	-1.67	1.44
		△Metric x IR	.27	.09	2.95	**.003**	.09	.45
		△Metric x CAT-index	-.03	.08	-.40	.69	-.20	.13
		IR x CAT-index	.005	.08	.06	.95	-.15	.16
		△Metric x IR x CAT-index	.03	.08	.32	.75	-.14	.19
	ATTA-index	△Metric	1.38e-05	.09	0	1	-.17	.17
		IR	.008	.08	.11	.92	-.15	.16
		ATTA-index	.02	.81	.02	.98	-1.58	1.61
		△Metric x IR	.33	.09	3.51	**4.57e-04**	.14	.51
		△Metric x ATTA-index	.04	.08	.50	.61	-.12	.20
		IR x ATTA-index	.02	.08	.27	.79	-.13	.18
		△Metric x IR x ATTA-index	.18	.08	2.25	**.02**	.02	.34
Efficiency	CAT-dist	△Metric	.15	.11	1.45	.15	-.05	.36

	SD	-.01	.03	-.38	.71	-.06	.04
	CAT-dist	-.01	.89	-.01	.99	-1.76	1.74
	∆Metric x SD	.09	.03	2.76	**.01**	.03	.16
	∆Metric x CAT-dist	-.17	.12	-1.50	.13	-.40	.05
	SD x CAT-dist	.01	.03	.31	.76	-.05	.06
	∆Metric x SD x CAT-dist	-.05	.04	-1.32	.19	-.12	.02
CAT-index	∆Metric	.16	.11	1.51	.13	-.05	.38
	SD	-.01	.03	-.36	.72	-.06	.04
	CAT-index	-.10	.91	-.11	.91	-1.88	1.68
	∆Metric x SD	.09	.03	2.78	**.01**	.03	.16
	∆Metric x CAT-index	.14	.10	1.31	.19	-.07	.34
	SD x CAT-index	-.002	.02	-.10	.94	-.05	.05
	∆Metric x SD x CAT-index	.02	.03	.79	.43	-.04	.08
ATTA-index	∆Metric	.14	.11	1.31	.19	-.07	.35
	SD	-.01	.03	-.26	.79	-.06	.04
	ATTA-index	.08	.93	.09	.93	-1.74	1.91
	∆Metric x SD	.09	.03	2.62	**.009**	.02	.15
	∆Metric x ATTA-index	-.14	.10	-1.39	.16	-.35	.06
	SD x ATTA-index	-.003	.03	-.12	.91	-.05	.05
	∆Metric x SD x ATTA-index	-.05	.03	-1.77	.08	-.11	.01

Table S8. The significant association of SemNet changes and problem-solving remained significant when adding individual creative measures in the models. ∆Metric represents the difference between the considered metric between the PreSemNet and the PostSemNet. *Impact rating* (IR) corresponds to the importance of edges or nodes for solving the problem based on independent assessment. *Semantic distance* (SD) represents the semantic remoteness of edges or nodes based on all participants' baseline pre-RJT before the presentation of the problem. We ran a mixed model for each metric to predict problem-solving. In these models, we included creativity measures that showed significant correlation with problem-solving abilities (*CAT-dist*, *CAT-index*, or *ATTA-index*), and considered only metrics for which significant associations were found between SemNet changes and problem-solving in Study 1. Significant results ($p < .05$) are in bold.

			β	SE	z value	p value	CI 95%
Weight	isSolved	△Metric	-.06	.06	-.93	.35	-.18 .06
		IR	-.001	.03	-.06	.95	-.05 .05
		SD	.03	.03	1.25	.21	-.02 .08
		△Metric x IR	-.02	.03	-.74	.46	-.07 .03
		△Metric x SD	.01	.03	.31	.75	-.05 .07
		IR x SD	.05	.03	2.02	.04	.001 .10
		△Metric x IR x SD	-.03	.03	-.99	.32	-.09 .03
	isTrans	△Metric	.001	.05	.02	.98	-.10 .11
		IR	.01	.02	.47	.64	-.03 .05
		SD	-.001	.02	-.04	.96	-.04 .04
		△Metric x IR	-.01	.02	-.66	.51	-.05 .03
		△Metric x SD	-.05	.02	-2.05	**.04**	-.09 -.002
		IR x SD	-.03	.02	-1.35	.18	-.07 .01
		△Metric x IR x SD	.01	.02	.29	.77	-.04 .05
Efficiency	isSolved	△Metric	-.07	.09	-.77	.44	-.24 .10
		IR	-.01	.03	-.44	.66	-.06 .04
		SD	.04	.03	1.53	.13	-.01 .09
		△Metric x IR	-.03	.03	-1.00	.32	-.08 .03
		△Metric x SD	-.02	.03	-.82	.41	-.08 .03
		IR x SD	.05	.03	2.07	.04	.003 .11
		△Metric x IR x SD	-.05	.03	-1.53	.13	-.10 .01
	isTrans	△Metric	.07	.08	.89	.38	-.08 .22
		IR	.02	.02	.87	.39	-.02 .06
		SD	.005	.02	.26	.80	-.03 .04
		△Metric x IR	-.001	.02	-.05	.96	-.04 .04
		△Metric x SD	.005	.02	.22	.82	-.04 .05
		IR x SD	-.03	.02	-1.25	.21	-.07 .01
		△Metric x IR x SD	.01	.02	.41	.68	-.04 .06
Clustering coefficient	isSolved	△Metric	-.01	.44	-.01	.99	-.88 .87
		IR	.03	.10	.34	.74	-.16 .23
		SD	.03	.10	.34	.73	-.16 .23
		△Metric x IR	-.03	.14	-.19	.85	-.30 .24
		△Metric x SD	.14	.15	.95	.34	-.15 .42
		IR x SD	.08	.10	.79	.43	-.12 .27
		△Metric x IR x SD	-.12	.15	-.82	.42	-.41 .17
	isTrans	△Metric	-.35	.51	-.69	.49	-1.35 .64
		IR	.07	.08	.85	.40	-.09 .23
		SD	.04	.08	.47	.64	-.13 .20
		△Metric x IR	-.16	.13	-1.23	.22	-.41 .09
		△Metric x SD	-.08	.13	-.57	.57	-.34 .19
		IR x SD	.14	.08	1.66	.10	-.02 .30

		∆Metric x IR x SD	-.17	.14	-1.22	.22	-.44	.10
Eigenvector centrality	isSolved	∆Metric	.04	.08	.49	.63	-.12	.19
		IR	-.01	.08	-.09	.93	-.16	.15
		SD	-.02	.08	-.21	.83	-.17	.14
		∆Metric x IR	-.05	.08	-.63	.53	-.20	.10
		∆Metric x SD	-.04	.08	-.45	.65	-.19	.12
		IR x SD	.05	.08	.65	.52	-.10	.20
		∆Metric x IR x SD	-.11	.08	-1.38	.17	-.27	.05
	isTrans	∆Metric	-.02	.06	-.35	.73	-.14	.10
		IR	-.001	.06	-.02	.99	-.12	.12
		SD	.0004	.06	.01	1	-.12	.12
		∆Metric x IR	-.01	.06	-.20	.84	-.13	.10
		∆Metric x SD	-.03	.06	-.58	.56	-.15	.08
		IR x SD	.001	.06	.02	.99	-.12	.12
		∆Metric x IR x SD	.005	.06	.08	.94	-.11	.12

Table S9. Association between SemNet changes and problem-solving was specific to the problem. ∆Metric represents the difference between the considered metric between the PreSemNet and the PostSemNet. *Impact rating* (IR) corresponds to the importance of edges or nodes for solving the problem based on independent assessment. *Semantic distance* (SD) represents the semantic remoteness of edges or nodes based on all participants' baseline pre-RJT before the presentation of the problem. We ran a mixed model for each metric to predict solving of the second problem in naive condition (isSolved) or its analogous problem (isTrans, see Figure S5). Significant results ($p < .05$) are in bold.

Supplementary Notes

Supplementary Note 1: Relationship between problem-solving and creative abilities

Creativity tasks

During the 4h-experiment, participants completed several creativity tasks. All tasks were computed using the Psychopy software [1] running on individual computers (except for the ATTA that used paper and pencil material) in a classroom dedicated to cognitive experiments. Task-related instructions were explained before the start of each task.

The Combined Associates Task (CAT) [2,3]

The CAT consists in finding a word connecting three given unrelated cue words. Semantic association strength between the expected solution and the three cue words was controlled for each trial. Hence, distant trials (i.e., trials with a distant solution) and close trials (i.e., trials with a close solution) were built. According to the associative theory of creativity, the more remote the elements to be combined (i.e., distant trials), the more creative the process.

Participants underwent 40 trials (20 distant trials and 20 close trials) in random order. They had up to 30 seconds to write their answer with the keyboard. To explore individual creative abilities, based on [2,3], we considered the number of correct responses in distant trials (*CAT-dist*) and computed a *CAT-index* (corresponding to the difference between performance on close and distant trials, divided by the mean performance in both conditions). The *CAT-index* reflects the ability to solve distant trials (the more creative condition) when controlling for performance in the less creative condition (close trials): the lower the *CAT-index*, the higher the creativity abilities.

The Abbreviated Torrance Test for Adult (ATTA) [4]

The ATTA includes three activities (one verbal and two figural). In the first activity, participants had to generate as many ideas as they could for an open-ended problem ("Suppose that you can walk on air or fly. Imagine what problem you might encounter"). In the second activity, participants were asked to draw pictures as unusual as possible by using two incomplete figures. In the last activity, participants had to draw as many pictures as they could using triangles.

Participants were asked to perform each activity one at a time with a pencil on a dedicated piece of paper. They had 3 minutes per activity. Before starting, instruction was read orally to participants. According to the ATTA manual, participants were evaluated on their productions' fluency, originality, elaboration, flexibility, and other indicators. A composite index (*ATTA-index*) was computed based on all scaled scores: the higher the *ATTA-index*, the higher the creativity abilities.

The Inventory of Creative Activities and Achievements (ICAA) [5]

The ICAA is a self-questionnaire assessing creative activities and achievements across eight real-life creative domains (e.g., literature, music, art and craft, cooking, sport, visual art, performing arts, science, and engineering). The creative activities (*C-Act*) score reflects the frequency in which participants engaged in various creative activities. Six different questions were asked for each domain, and participants reported the frequency with which they engaged in each activity during the last ten years, using a scale ranging from 0 (never) to 4 (more than ten times). For each participant, the final domain-general score of *C-Act* was the sum of the creative activities across all activities of the eight different domains. The creative achievements (*C-Ach*) score estimated the level of achievement reached in all creative domains. Ten different

levels of achievement were included for each domain going from 0 (never engaged in this domain) to 10 (I have already sold some of my work in this domain). For each participant, the final domain-general score of *C-Ach* was the sum of the scores across the eight different domains.

Creative problem-solving assessed by our riddles relates to creative abilities

To assess how much our riddles represent creative problems, we explored the relationship between the ability to solve the four riddles (i.e., the individual solving rate across the four riddles) and behavioral variables of creativity tasks. Relations were assessed with several Spearman correlations, corrected for multiple comparisons with Bonferroni correction. Correlations that survived the correction needed to maintain their p value under .01 (= 0.05/5).

We found that riddles' solving rate was positively and significantly correlated with the *CAT-dist* (rho = .33, p = 9.15 10^{-4}), the *ATTA-index* (rho = .29, p = 3.84 10^{-3}). It was negatively and significantly correlated with the *CAT-index* (rho = -.38, p = 1.38 10^{-4}; Figure S4). The correlations between riddle solving and *C-Ach* (rho = 0.20, p = .052) and *C-Act* (rho = 0.09, p = .35) were not statistically significant.

These results strongly suggest that our riddles' material measures creative thinking and refers to creative problem-solving.

Individual creative abilities did not significantly impact the link between SemNet changes and problem-solving

To ensure that the significant relationships that we found between SemNet changes and problem-solving were not influenced by individual creative abilities, we provided additional control analyses. For the SemNet changes showing significant interaction effect of ΔMetric x *impact rating* (IR) or ΔMetric x *semantic distance* (SD) on problem-solving, we tested whether these interaction effects remained significant after adding individual creative measures (Creativity) in the models. We used creative measures that previously showed a significant correlation with problem-solving abilities (i.e., *CAT-dist*, *CAT-index*, and *ATTA-index*). The models can be formalized as follow:

(7) isSolved = $\boldsymbol{\beta}_1$ x ΔMetric + $\boldsymbol{\beta}_2$ x IR + $\boldsymbol{\beta}_3$ x Creativity + $\boldsymbol{\beta}_4$ (ΔMetric x IR) + $\boldsymbol{\beta}_5$ (ΔMetric x Creativity) + $\boldsymbol{\beta}_6$ (IR x Creativity) + $\boldsymbol{\beta}_7$ (ΔMetric x IR x Creativity) + (1|Subject) + (-1+ΔMetric|Subject) + k

(8) isSolved = $\boldsymbol{\beta}_1$ x ΔMetric + $\boldsymbol{\beta}_2$ x SD + $\boldsymbol{\beta}_3$ x Creativity + $\boldsymbol{\beta}_4$ (ΔMetric x SD) + $\boldsymbol{\beta}_5$ (ΔMetric x Creativity) + $\boldsymbol{\beta}_6$ (SD x Creativity) + $\boldsymbol{\beta}_7$ (ΔMetric x SD x Creativity) + (1|Subject) + (-1+ΔMetric|Subject) + k

In Table S8, we report all statistical results. We were particularly interested in the ΔMetric by *impact rating* (or *semantic distance*) interaction effect when adding the creative measure in the model. We found that the interaction effect of ΔMetric and *impact rating* (IR) or ΔMetric and *semantic distance* (SD) on problem-solving remained significant after adding individual creative measures (*weight*: *CAT-dist*, $\boldsymbol{\beta}$ = .07, p = .01, CI95 = [.02;.13]; *CAT-index*, $\boldsymbol{\beta}$ = .07, p = .01, CI95 = [.02;.13]; *ATTA-index*: $\boldsymbol{\beta}$ = .07, p = .01, CI95 = [.02:.13]; *efficiency*: *CAT-dist*, $\boldsymbol{\beta}$ = .08, p = .004, CI95 = [.03;.14]; *CAT-index*, $\boldsymbol{\beta}$ =.08, p = .003, CI95 = [.03;.14]; *ATTA-index*, $\boldsymbol{\beta}$ = .08, p = .004, CI95 = [.03;.14]; *eigenvector centrality*: *CAT-dist*, $\boldsymbol{\beta}$ = .28, p = .002, CI95 = [.11;.46]; *CAT-index*, $\boldsymbol{\beta}$ = .27, p = .003, CI95 = [.09;.45]; *ATTA-index*, $\boldsymbol{\beta}$ = .33, p = 4.57 10^{-4}, CI95 = [.14;.51]; *efficiency*: CAT-dist, $\boldsymbol{\beta}$ = .09, p = .01, CI95 = [.03;.16]; *CAT-*

index, **β** = .09, *p* = .01, CI95 = [.03;.16]; *ATTA-index*, **β** = .09, *p* = .009, CI95 = [.02;.15]). In addition, we observed a significant interaction effect of ∆Metric, *impact rating*, and *ATTA-index* on problem-solving for *eigenvector centrality* (**β** = 0.18, *p* = .02, CI95 = [.02;.34]).

These results suggest that the significant relationships we observed between SemNets changes and problem-solving were not merely due to individual differences in creative abilities. In addition, we found a significant three-way interaction effect of ∆Metric, *impact rating*, and *ATTA_index* on problem-solving. It suggests that the effect of solution-based SemNet changes (solution-based nodes becoming more central) on successful solving was higher for individual with lower divergent thinking abilities (Figure S7), which could be counterintuitive. However, the two-way interaction effect between ∆Metric and *impact rating* on problem-solving stayed significant even when the three-way interaction between *ATTA-index*, ∆Metric, and *impact rating* on problem-solving was. We could speculate that problem-solving requires a balance between divergent thinking (to generate alternative problem representation and solutions) and convergent thinking (to respect constraints related to the problem statement). This hypothesis is supported by previous study showing that RAT solving requires a divergent and then a convergent process [6] or other suggesting that divergent thinking and convergent problem-solving are not strongly related [7].

Supplementary Note 2: Analogical transfer and problem-solving

We explored if the solving rate of each riddle taken individually differed between the *naive* condition and the *transfer* conditions using chi-square analyses (corrected with Yates method if needed).

We found that the solving rate in the *transfer* condition was significantly higher than in the *naive* condition for the Car riddle (42.6% and 18.3% respectively, $\chi^2(1) = 6.66$, $p = 9.88\ 10^{-3}$), and the Daniel riddle (28% and 8.2% respectively, $\chi^2(1) = 6.55$, $p = .01$), but it was not statistically the case for the Zoe riddle (46% and 28.6% respectively, $\chi^2(1) = 3.21$, $p = .07$), and the Bar riddle (18.2% and 6.7% respectively, $\chi^2(1) = 2.72$, $p = .10$) (Table S7).

In summary, for each riddle taken separately, we found a significant analogical transfer effect on problem-solving in two riddles and a statistical tendency for the two remaining riddles. It may suggest that considering several riddles could increase the sensitivity to capture an analogical transfer effect on problem-solving.

Supplementary Note 3: Confidence in responses and attentional focus on the riddle

We compared the average confidence rating across participants when a response was correct (when relevant) vs. when responses were incorrect (that is, the average confidence of all incorrect responses at the individual level) for all riddles together using two-tailed paired-samples t-test analyses. Confidence levels of participants' responses were significantly higher for correct responses (mean = 75.19, SEM = 1.82) than for incorrect ones (mean = 35.38, SEM = 1.53, $t(214) = 8.66$, $p < .001$).

During the solving phase, the proportion of participants who reported being focused on the task (i.e., attempting to solve the riddle) remained above 50% for each probe, suggesting that participants were mostly engaged in solving the riddle.

Supplementary Note 4: SemNet organization of solvers and non-solvers was not significantly different at baseline

To ensure that the difference in changes of SemNets between the solver and non-solver group was not driven by inter-group differences at baseline, we tested whether the two groups had similar SemNet organization before the presentation of the problem (Pre-RJT). We used analyses of variance (ANOVA) to compare the $\text{Metric}_{\text{preSemNet}}$ as a function of the group (solver or non-solver). Participants were added as a random factor in the model. We repeated the model for each metric that we calculated (*weight*, *efficiency*, *clustering coefficient*, and *eigenvector centrality*), and for each different riddle.

Study 1

The $\text{Metric}_{\text{preSemNet}}$ was not statistically different between the solvers and non-solvers for Zoe riddle (*weight*: $F(1,34) = 0.006, p = .94$; *efficiency*: $F(1,34) = 0.34, p = .56$; *clustering coefficient*: $F(1,34) = 0.01, p = .92$; *eigenvector centrality*: $F(1,34) = 1.67, p = .21$), Car riddle (*weight*: $F(1,36) = 0.16, p = .69$; *efficiency*: $F(1,36) = 0.50, p = .49$; *clustering coefficient*: $F(1,36) = 0.15, p = .70$; *eigenvector centrality*: $F(1,36) = 1.32, p = .26$), Daniel riddle (*weight*: $F(1,32) = 0.18, p = .67$; *efficiency*: $F(1,32) = 0.09, p = .77$; *clustering coefficient*: $F(1,32) = 0.79, p = .38$; *eigenvector centrality*: $F(1,32) = 1.58, p = .22$), and Bar riddle (*weight*: $F(1,32) = 0.27, p = .61$; *efficiency*: $F(1,32) = 1.01, p = .32$; *clustering coefficient*: $F(1,32) = 0.01, p = .93$; *eigenvector centrality*: $F(1,32) = 0.28, p = .60$).

Overall, the SemNet properties of efficiency, clustering, and centrality did not statistically differ between solvers and non-solvers at baseline.

Study 2

The $\text{Metric}_{\text{preSemNet}}$ was not statistically different between the solvers and non-solvers for all metrics (*weight*: $F(1,116) = 0.006, p = .94$; *efficiency*: $F(1,116) = 0.04, p = .84$; *clustering coefficient*: $F(1,116) = 0.07, p = .80$; *eigenvector centrality*: $F(1,116) = 0.04, p = .84$).

Overall, the SemNet properties of efficiency, clustering, and centrality did not statistically differ between solvers and non-solvers at baseline.

Study 3

The $\text{Metric}_{\text{preSemNet}}$ was not statistically different between the solvers of Zoe riddle combined from the three experiments and the solution group in Study 3 (who was given the solution after failing to solve the riddle themselves) for any metric (*weight*: $F(1,70) = 1.75, p = .19$; *efficiency*: $F(1,70) = 1.47, p = .23$; *clustering coefficient*: $F(1,70) = 2.03, p = .16$; *eigenvector centrality*: $F(1,70) = 0, p = .99$).

Overall, the SemNet properties of efficiency, clustering, and centrality did not statistically differ between the solver and the solution groups.

Supplementary Notes 5: Association between SemNet changes and problem-solving was specific to the problem

To ensure that the relationship that we found between SemNets changes and problem-solving was a specific effect of the problem in hand (and not reflecting the ability to solve any unrelated riddle), we ran additional control analyses. We investigated if SemNets changes related to a given problem were associated with the solving of other problems that showed no analogical similarities with it. The design of Study 1 allowed us to explore this question. We tested whether the SemNets changes related to the first riddle that participants encountered were associated with the solving of each one of the unrelated second couple of riddles that the participants were asked to solve later (Figure S5).

We used nonlinear mixed-effect models to assess if restructuring (ΔMetric), *impact rating* (IR) and *semantic distance* (SD) related to one riddle were associated with solving another riddle in *naive* condition (isSolved) or in *transfer* condition (isTrans). The models can be formalized as follow:

(9) $\text{isSolved} = \boldsymbol{\beta}_1 \times \Delta\text{Metric} + \boldsymbol{\beta}_2 \times \text{IR} + \boldsymbol{\beta}_3 \times \text{SD} + \boldsymbol{\beta}_4 (\Delta\text{Metric} \times \text{IR}) + \boldsymbol{\beta}_5 (\Delta\text{Metric} \times \text{SD}) + \boldsymbol{\beta}_6 (\text{IR} \times \text{SD}) + \boldsymbol{\beta}_7 (\Delta\text{Metric} \times \text{IR} \times \text{SD}) + (-1+\Delta\text{Metric}|\text{Subject}) + k$

(10) $\text{isTrans} = \boldsymbol{\beta}_1 \times \Delta\text{Metric} + \boldsymbol{\beta}_2 \times \text{IR} + \boldsymbol{\beta}_3 \times \text{SD} + \boldsymbol{\beta}_4 (\Delta\text{Metric} \times \text{IR}) + \boldsymbol{\beta}_5 (\Delta\text{Metric} \times \text{SD}) + \boldsymbol{\beta}_6 (\text{IR} \times \text{SD}) + \boldsymbol{\beta}_7 (\Delta\text{Metric} \times \text{IR} \times \text{SD}) + (-1+\Delta\text{Metric}|\text{Subject}) + k$

In Table S9, we report all statistical results. We found (i) no statistically significant interaction effect of ΔMetric and *impact rating* on solving another problem in *naive* condition (*weight*: **β** = -0.02, p = .46, CI95 = [-.07;.03]; *efficiency*: **β** = -0.03, p = .32, CI95 = [-.08;.03]; *clustering coefficient*: **β** = -0.03, p = .85, CI95 = [-.30;.24]; *eigenvector centrality*: **β** = -0.05, p = .53, CI95 = [-.20;.10]), (ii) no statistically significant interaction effect of ΔMetric and *impact rating* on solving another problem in *transfer* condition (*weight*: **β** = -0.01, p = .51, CI95 = [-.05;.03]; *efficiency*: **β** = $-1.17\ 10^{-3}$, p = .96, CI95 = [-.04;.04]; *clustering coefficient*: **β** = -0.16, p = .22, CI95 = [-.41;.09]; *eigenvector centrality*: **β** = -0.01, p = .84, CI95 = [-.13;.10]), and (iii) no statistically significant interaction effect of ΔMetric and *semantic distance* on solving another problem in *naive* condition (*weight*: **β** = $9.13\ 10^{-3}$, p = .75, CI95 = [-.05;.07]; *efficiency*: **β** = -0.02, p = .41, CI95 = [-.08;.03]; *clustering coefficient*: **β** = 0.14, p = .34, CI95 = [-.15;.42]; *eigenvector centrality*: **β** = -0.04, p = .65, CI95 = [-.19;.12]). We found a significant interaction effect of ΔMetric and *semantic distance* on solving another problem in the *transfer* condition for *weight* (**β** = -0.05, p = .04, CI95 = [-.09;-.002]), suggesting that higher remoteness-based restructuring was conducive to lower solving (opposite direction compared to the prediction of transfer on a true analogy). However, this significant result did not survive the correction for multiple comparisons for non-independent tests (see Method). The interaction effect of ΔMetric and *semantic distance* on solving another problem in *transfer* condition was not statistically significant for other metrics (*efficiency*: **β** = $5.23\ 10^{-3}$, p = .82, CI95 = [-.04;.05]; *clustering coefficient*: **β** = -0.08, p = .57, CI95 = [-.34;.19]; *eigenvector centrality*: **β** = -0.03, p = .56, CI95 = [-.15;.08]).

These results suggest that problem-related restructuring was significantly associated with solving the considered problem or an analogous one, but not statistically associated with solving unrelated riddles.

Supplementary References

1. Peirce, J. *et al.* PsychoPy2: Experiments in behavior made easy. *Behavior research methods* **51**, 195–203 (2019).
2. Bendetowicz, D., Urbanski, M., Aichelburg, C., Levy, R. & Volle, E. Brain morphometry predicts individual creative potential and the ability to combine remote ideas. *Cortex* **86**, 216–229 (2017).
3. Bendetowicz, D. *et al.* Two critical brain networks for generation and combination of remote associations. *Brain* **141**, 217–233 (2018).
4. Torrance, E. P. Predictive Validity of the Torrance Tests of Creative Thinking*. *The Journal of Creative Behavior* **6**, 236–262 (1972).
5. Diedrich, J. *et al.* Assessment of real-life creativity: The Inventory of Creative Activities and Achievements (ICAA). *Psychology of Aesthetics, Creativity, and the Arts* **12**, 304 (2018).
6. Smith, K. A., Huber, D. E. & Vul, E. Multiply-constrained semantic search in the Remote Associates Test. *Cognition* **128**, 64–75 (2013).
7. Reiter-Palmon, R. & Arreola, N. J. Does Generating Multiple Ideas Lead to Increased Creativity? A Comparison of Generating One Idea vs. Many. *Creativity Research Journal* **27**, 369–374 (2015).
